# Making and re-making the hospital: Using collaging and mapping to explore children and young people’s experiences of the hospital environment

**DOI:** 10.1177/26349795251363044

**Published:** 2025-08-01

**Authors:** Rebecka Fleetwood-Smith

**Affiliations:** 1980 University of Bristol, UK

**Keywords:** Multimodal approaches, creative research methods, senses, atmospheres, paediatric hospitals

## Abstract

Paediatric hospital settings are complex environments catering for children and young people, from birth up until early adulthood. Research that focuses on these settings often involves concentrating on specific design elements and features rather than considering the sensory, embodied, and atmospheric qualities of these environments. This project aims to explore the past, present, and future of the NHS hospital focusing on the sensory environment. Research consisted of two strands of study: (1) archival and oral history research; and (2) creative research, working at sites with patients, staff, and visitors. Using collaging and mapping, this study worked with children and young people accessing outpatient auditory/visual services to explore their hopes and wants for the hospital environment. This research demonstrates the opportunities that different modes of expression afford in exploring the experiences of children and young people, and highlights the importance of this when working with people with sensory impairments. Findings demonstrate the sensitive and considered ways in which children and young people make and re-make places in the hospital to produce private, creative, and imaginative spaces.

## Introduction

This study involved working with children and young people who were outpatients accessing services for visual and/or auditory impairments. This forms part of ‘Sensing Spaces of Healthcare' (SSOH) – a UKRI funded project (2020–2027) (Hospital Senses n.d.), led by Dr Victoria Bates. The project is delivered in partnership with hospital arts organisations Fresh Arts, North Bristol NHS Trust (NBT), and GOSH Arts, Great Ormond Street Hospital, London (GOSH). The respective hospital arts organisations lead live arts programmes, hold art collections, curate exhibitions, and commission art and design to enhance the built environment.

SSOH aims to explore the past, present, and future of the NHS hospital environment. Research consists of two strands of study: (1) archival and oral history research and; (2) creative research working at sites with patients, staff, and visitors. This article focuses on research carried out during the creative strand of study across 2020–2024. This phase involved working with NHS patients, staff, and visitors to explore sensory experiences within hospital environments and identify opportunities for change within these settings. The research was underpinned by a sensory, embodied, and creative approach to study. Thematic research findings informed the development of a series of prototypes installed at the respective hospital sites. The prototypes are currently under study (2024–2027).

The aims of this article are twofold: (1) to illuminate the opportunities that creative methods, which are inherently multimodal, hold when working with children and young people accessing outpatient auditory/visual services and; (2) to demonstrate the sensitive, considered and creative ways that children and young people make and re-make the hospital environment. This article will be of interest to multimodal scholars due to its sensory and creative focus, its use of collaging and map-making as routes to meaning-making with children and young people living with auditory/visual impairments, and its exploration of the atmospheres that emerged during the creative research.

Supporting children and young people to participate in research is challenging and involves navigating methodological and ethical complexities that are exacerbated when working in healthcare settings. Recent increased emphasis on including patients and the public in the planning, design, and management of healthcare services includes children and young people (Lambert et al., 2014). As Bates (2025) highlights, the design of children’s hospitals increasingly involves collaboration or consultation with children, young people, and their families to centre their experiences, and these processes mirrors those carried out with adults.

Children and young people have clear ideas about the design of paediatric hospitals. Scaling down elements relative to children’s proportions (Adams et al., 2010); bright colours (Coad and Coad, 2008); and ‘emblems of childhood such as teddy bears and balloons' (Blumberg and Devlin, 2006:293) have been rejected by children and young people in existing research. Children and young people are concerned about age-associated characteristics of hospital spaces (Birch et al., 2007; McKenzie et al., 2010) and demonstrate sensitivity to what the environments offer them and their families (Payam et al., 2023). Such considerations highlight the complexities involved in designing spaces that must cater for newborns to young adults (in the UK the exact age is dependent on the hospital and the individual’s treatment) and their families (Cartland et al., 2018).

McLaughlan and Willis (2021) propose ‘atmospheric inclusiveness’ as a critical design aspiration for paediatric hospitals. The turn to ‘atmospheres’ in recent years draws upon and extends literature on affect that involves thinking about ‘the relationship between bodies and spaces to attend to the often-taken-for-granted and implicit effects that encounters between human and non-human bodies generate’ (Anderson and Ash, 2015: 34). McLaughlan and Willis (2021) use ‘atmospheres’ to refer to how spatial properties, light, exterior views, materials, artworks, and visual cues contribute to how the environment feels. They claim that people do not encounter design features in isolation; rather, environments are perceived as a whole.

Stewart (2011) similarly proposes analytic attention to atmospheres and presents a series of cases to illuminate how things matter ‘not because of how they are represented but because they have qualities, rhythms, forces, relations, and movements.’ Stewart’s (2011: 446) case on ‘pockets’ is of particular interest with regards to this research.

There's a pause, a temporal suspension animated by the sense that something is coming into existence. The subject is called to a state of attention that is also an impassivity a watching and waiting, a living through, an attunement to what might wind up or snap into place. Events and outcomes are immanent, unknown but pressing… The unfolding of a pocket slowed and amplified to see what might be in it.

The notion of ‘pockets’ within the study of atmospheres can support the exploration of specific moments in hospital environments, suggesting how something may unfold, come into existence, or slip away. This is of great interest when considering how children and young people encounter hospital spaces.

Sumartojo and Pink (2018) caution that atmospheres cannot be designed and illuminate how design interventions ‘make possible the circumstances through which particular types of atmospheres might emerge’ (2018: 95). Atmospheres are irreducible; they are more than the sum of their parts and morph and shift accordingly. Attending to the conditions in which they ‘emerge and circulate’ (Sumartojo et al., 2020: 26) can allow us to interrogate the design of the hospital and the interventions that contribute to specific atmospheres. For instance, different sensory encounters – what a setting looks like, the different materials, sounds, and smells encountered – and the people in those spaces all shape the atmospheres that come into being. Attending to atmospheres allows us to examine the unfixed and ever-changing nature of hospital environments, and how design is also ongoing (Sumartojo et al., 2020; Sumartojo and Pink, 2018). Understanding the built environment in flux necessitates a shift in thinking about how settings are encountered and used, and how they may be enhanced.

This study, through attending to the atmospheric, sensory and emotional, builds upon the growing area of research that centres the experiences of children and young people within hospital design. The methods used demonstrate how research approaches that promote different modes of expression can support children and young people with different access needs to be active research participants, whose ideas and creativity are key in shaping hospital environments of the future.

## Methods

The research presented here used multimodal methods to explore children and young people’s experiences of accessing outpatient visual and/or auditory services at Great Ormond Street Hospital, London (GOSH). GOSH is an internationally recognised centre for treating children, who often have longstanding relationships with the hospital. This research was situated in a newly opened building designed specifically for auditory/visual services. This provided an opportunity to explore people’s experiences on encountering the new space. This research was not an attempt to carry out a post-occupancy evaluation – a process which involves analysing the function and performance of a building in use. Rather, this research considered what is important to children and young people, identifying opportunities for change. Findings from this research were used to develop a series of prototypes, currently under study at the study sites (2024–2027).

### Developing the creative research approaches

Traditionally qualitative methods have failed to account for the visual and sensory and often fail to attend to bodily experiences (Jewitt and Leder Mackley, 2019). This research used methods that moved beyond the verbal and the visual to explore experiences in embodied ways, drawing upon creative and design-led approaches to research (Kara, 2020; Wallace et al., 2013; Woodward, 2020; Zino et al., 2021); multimodal approaches (Jewitt and Leder Mackley, 2019; Yamada- Rice et al., 2023); and sensory approaches (Pink, 2015). As Hackett (2015) notes, there is a need to develop research approaches that are relevant to children and that recognise the nuanced non-verbal ways in which they communicate.

Participatory and creative approaches are often advocated when working with children and young people and can illuminate their experiences within hospitals (see e.g. Birch et al., 2007; Gibson et al., 2010; Lambert et al., 2014). This research drew upon such approaches and foregrounded thinking through making (Ingold, 2013) to centre imaginative ways of working. This drew parallels with existing multimodal research. For example, Yamada-Rice et al. (2023) developed a play kit to support children undergoing magnetic resonance imaging (MRI) scans, hosting workshops in which children drew, created models, and played, to develop understanding.

Designing the methods involved working with the project’s hospital arts project partners (GOSH Arts and Fresh Arts), GOSH Young Person’s Advisory Group, and GOSH play specialists (see Fleetwood-Smith, 2021). The research attended to situated practices and experiences, which typically involves researchers working closely with research participants over time (see e.g. Duque et al., 2019). Due to restrictions during the pandemic, it was not possible to work with participants repeatedly or over a prolonged period, and methods were therefore designed to be achievable within a limited time frame, to be sensitive to the context of the pandemic, and to meet the possible access needs of participants.

I drew upon speculative design, which involves thinking critically about futures, while allowing for critique of, for instance, current settings (Tsekleves et al., 2019). During development with project partners and PPIE groups it was recommended that we use the term ‘dream hospital’ to minimise potential technocentric and temporal complexities associated with the word ‘futures’. The research methods were therefore designed to explore aspirations for future hospital environments, supporting a process of reflection to identify site-specific opportunities for change.

### Research methods in practice

The research methods used collaging and map-making (Fleetwood-Smith, 2021) employing a range of multisensory objects and materials to enable different ways of engaging and participating. The approaches drew upon existing methods and were informed by GOSH Arts’ participatory arts programme. Two approaches were designed for use on-site with children and young people:

1. *Collaging your Dream Hospital.* This research activity explored what participants’ dream hospital would feel like through abstract collaging. Different materials were provided to support the accessibility of the activity (details below).
2. *Mapping your Dream Hospital.* This research activity involved imagining multisensory encounters in participants’ dream hospital and sharing these on their Dream Hospital floor plan. Different materials were provided to support the accessibility of the activity (details below).

Adapting these methods to work with children (aged 3+) and young people living with hearing and/or visual impairments involved adopting multimodal strategies, considering the different ages of participants and how they might access and participate in the research. Reflexive notes are included here to situate the research methods in practice. My reflexive notes are shaped by my background and circumstances: I am a white, middle-class, non-disabled, cisgendered female who is an early career researcher/creative practitioner. Alongside my research role, I am a textile artist and lead creative workshops. My practice informs all aspects of my research process including, for instance, engagement with materials, the presentation of research activities, and creative facilitation.

**Figure 1.**
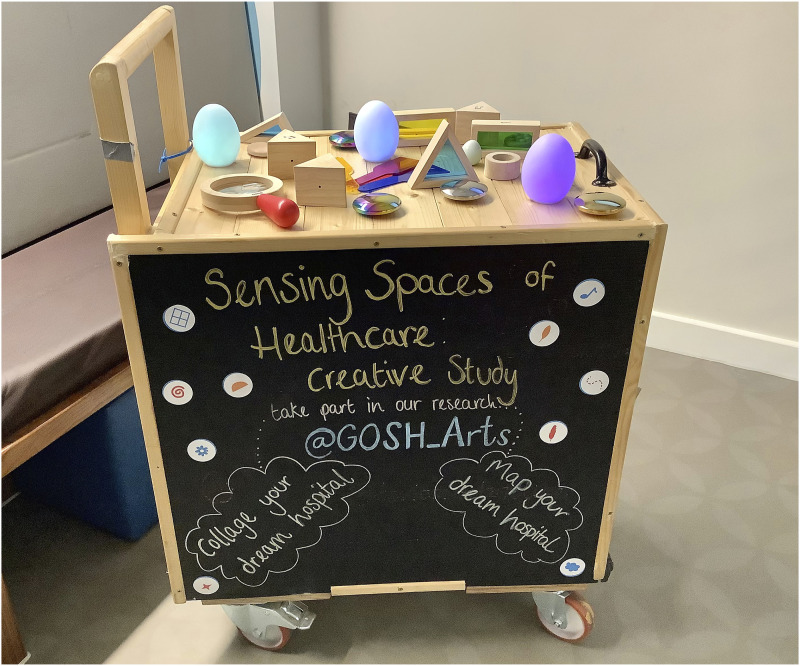
GOSH arts trolley used for the purpose of transporting research materials around the hospital. Objects were cleaned between use.

Figure 1 shows the GOSH Arts trolley repurposed to house different sensory objects that children and young people could use when participating in the research. Items included metallic discs that produced different sounds when shaken, coloured paddle lenses, building blocks, musical wooden shapes, tactile objects, and colour-changing glowing objects. The items were selected to be accessible and to support different forms of meaning-making. For example, participants could select, handle, sort, and place objects to form and express ideas.

Research was carried out in waiting areas before or after appointments, drawing upon GOSH Arts’ engagement work with children and young people. Participants chose whether to complete the collaging or mapping activity. Due to the partnership with GOSH Arts it was important that all children and young people could engage with the materials whether they chose to participate in the research or not. The following reflexive note illustrates some of the complexities involved in this way of working.

There were times when the waiting area was overwhelming. The space got quite busy and managing the trolley and materials/recruitment was really challenging. Originally, I had planned that I would work in almost a static way i.e. I would set up a table and then invite children/young people to work with me, but I typically moved to different waiting areas to work with children/young people/families. When working I often sat on the floor to be at the same level as the person that I was working with, and this could make looking after the trolley and materials difficult. My role was torn as on one hand I was a researcher and on the other hand I was offering creative engagement in the space. *Researcher Reflexive Note*

**Figure 2.**
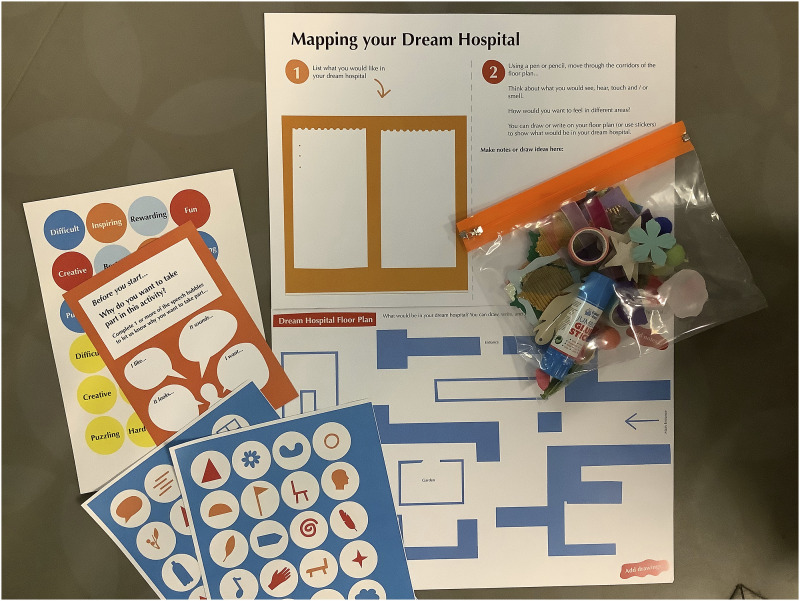
Mapping your dream hospital resources developed as singular packs to meet infection control measures.

Figure 2 shows the ‘Mapping your Dream Hospital’ resources developed for children and young people. Due to infection control measures, resources were produced as individual packs for both activities. Materials for ‘Mapping your Dream Hospital' consisted of:

- A3 sheet, with an initial ‘warm-up’ list making activity and instructions on mapping their dream hospital;
- A3 Dream Hospital Floorplan;
- A4 symbols and sticker sheets;
- Wallet of multisensory arts materials, including items recommended by GOSH YPAG and GOSH play team;
- A5 evaluation postcard, with an accompanying sticker sheet of words

The following reflexive note details the unanticipated ways of working that using the individual packs promoted.

An unexpected result of producing individual packs meant that some children/young people took their packs around the hospital with them. The outpatient service meant that they often had multiple appointments on the same day. Many participants initially worked with me to start their creative response and then continued working on this when waiting for the rest of their appointments. Once they had finished, they returned their responses to me describing what they had created, and we worked together to photograph it. In all cases children and young people took their completed physical activities home. Participants either approached the activities independently or with support from family members. Parents/guardians, with their intimate understanding of their child, often supported their involvement and, in some cases, this meant that parents/guardians almost became facilitators/co-researchers – this was not something that I had anticipated. *Researcher Reflexive Note*

**Figure 3.**
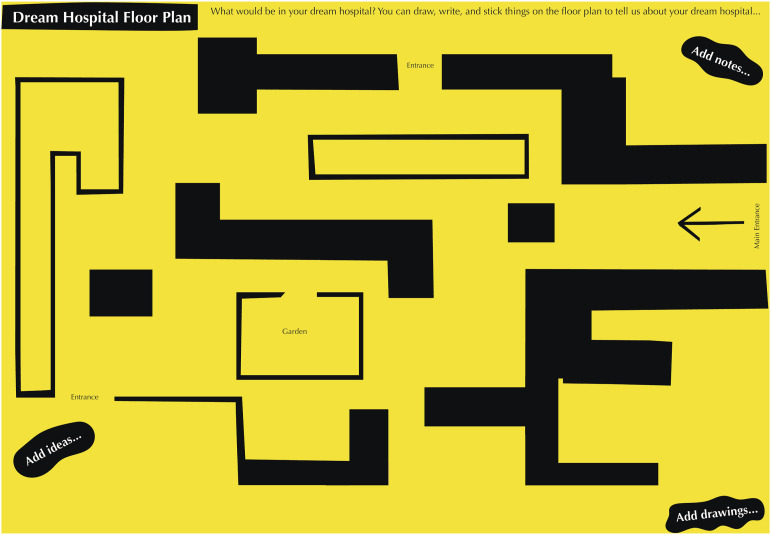
Dream Hospital Floor Plan in black and yellow colourway adapted for accessibility. Original illustration by Alex Higlett, Pirrip Press. Shared with permission.

The mapping activity was also resourced using large A1 maps (created in a yellow-and-black accessible colourway, see Figure 3) and I created tactile maps to ensure that participants with visual impairments could engage with the activity. Participants could interact with and place sensory objects on the maps to explore and form their ideas.

### Ethical considerations

Ethical approval for this research was granted by London Camden and Kings Cross NHS Research Ethics Committee (reference 21/LO/0045). Information was provided to families, and children and young people using written, visual, and verbal means. Parent/guardian consent was received for all participants and all participants agreed to take part in the research. Participants were recruited purposively, meaning that those accessing outpatient services were eligible to take part.

### Participants

Eight patients took part in this phase of research, all accessing outpatient services for auditory/visual impairments. One participant did not complete the demographic questionnaire, and not all participants completed all the questions. Of those who completed it, six identified as female and one as male. Participants identified as Asian/Asian British: Pakistani, Mixed/Multiple Ethnic Groups, White and Asian, White British, White English, and White and Polish and Asian. Where this information was collected, participants’ year of birth ranged from 2009–2017. Participants were not required to disclose the nature of their outpatient appointment(s) when taking part in the research. Codes are used to ensure confidentiality. Codes were selected in an attempt not to limit participants’ voices and experiences through an identity ascribed by a pseudonym.

## Data analysis

The analysis process involved an adapted reflexive thematic analysis approach (Braun and Clarke, 2019, 2021). The process involved a re-familiarisation with the data (reading and reviewing written and creative responses/interview transcripts and researcher notes), preliminary coding, grouping codes, developing initial themes, and refining themes. Data analysis focused on participants’ creative process (photographs of their collages and maps, and photographs of how they used the space), verbal responses, and written descriptions of what they had created. Analysis was ongoing and shaped by work throughout the project. My reflexive and creative practice implicitly informed the analysis process.

## Thematic findings

The following themes bring together insights from children and young people with my reflexive notes. The anonymised data consists of creative explorations made by participants and participant notes made during creative activity. The themes presented are connected, sitting under the major theme (1) ‘Making and re-making the hospital’ with the sub-themes (1.1) Attuning to atmospheric bubbles, (1.2) Crafting places in the hospital, and (1.3) Creativity and connection.

### Making and re-making the hospital (1)

‘Making and re-making the hospital’ speaks to the ways in which children and young people spoke about and demonstrated how they manipulated the hospital space, actively and physically changing the environment through the way they used spaces. This major theme acts as an umbrella for the sub-themes, encompassing the multifaceted ways in which children and young people used the hospital environment.

**Figure 4.**
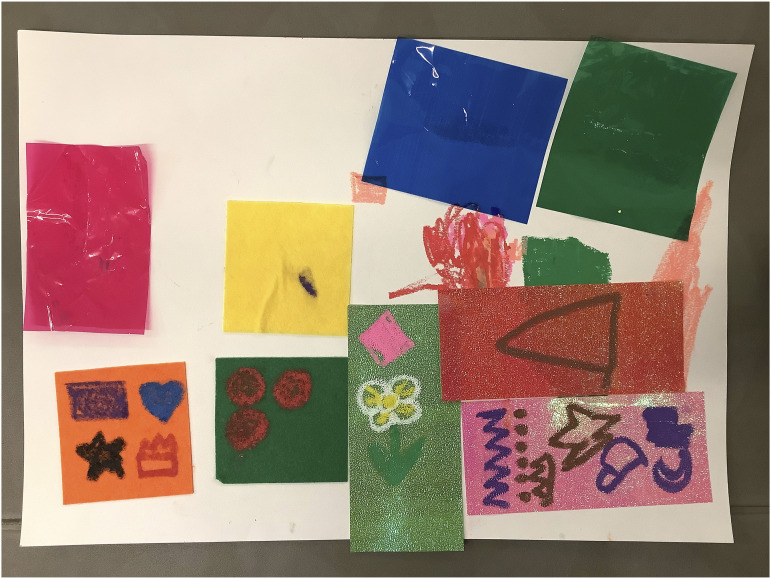
A2024’s collage of their dream hospital.

A2024’s collage (Figure 4) was created in sections. Their dream hospital was house-like and had ‘colourful windows, flowers, and lots of textures’ – each different colour and material was to be a different area of the hospital. A2024's collage expressed their want for different sensory elements within the hospital and for variation in the hospital environment.

**Figure 5.**
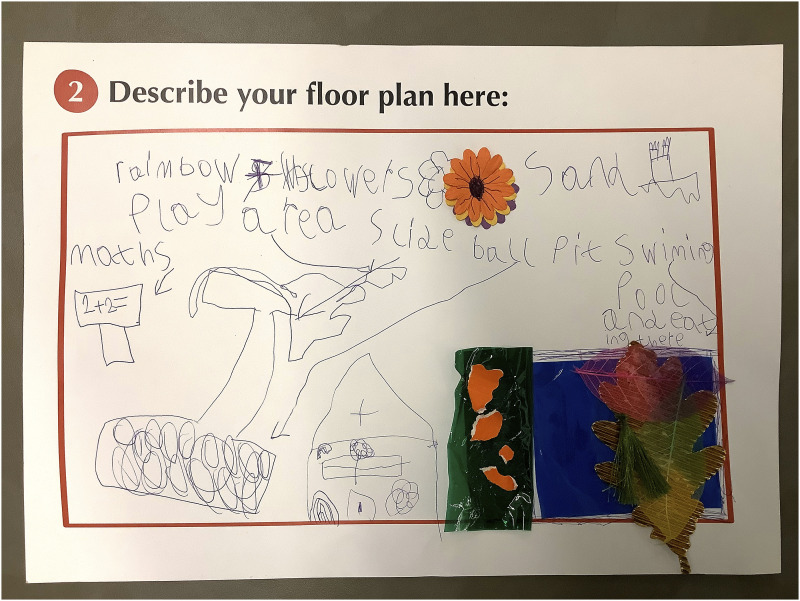
A2030’s collage of their dream hospital.

Similarly, A2030’s (Figure 5) dream hospital contained a house-like form, with different areas of the hospital, including spaces to play, eat, create, and study. The different areas demonstrate a want to be occupied by different things within the hospital.

The major theme ‘Making and re-making the hospital’ captures how children and young expressed a need for variety and distraction within the hospital environment. These elements are apparent throughout the following sub-themes.

### Attuning to atmospheric bubbles (1.1)

**Figure 6.**
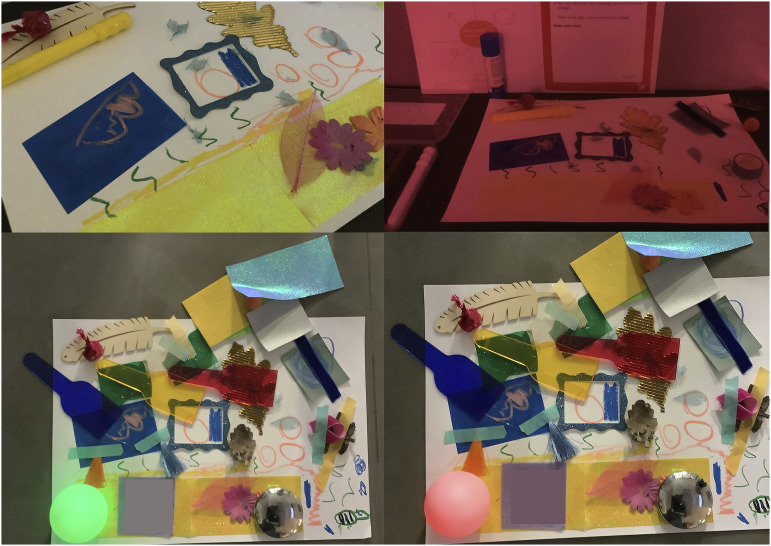
A2022 stages of creating their dream hospital collage.

‘Attuning to atmospheric bubbles’ draws upon reflexive notes and is inspired by a young child’s discussion of bubbles within the hospital. A2022 created a collage in which they drew bubbles, stating that they wanted to see bubbles in the hospital, as they find them calming. A2022 spoke of imagining a fish tank in the hospital in which the fish ate strawberries, and discussed wanting to play and watch the fish while waiting. A2022’s imaginative process, their want to create playful opportunities, and moments of calm and distraction could all translate to enhancing hospitals for children and young people. Their making process was iterative, and A2022 asked to photograph the many different versions of their collage, thus recording their selection, placement, and repositioning of various objects and materials (Figure 6). Their use of and engagement with objects, along with their drawings, and verbal descriptions, were all part of their meaning-making process.

To revisit and build upon Stewart’s (2011) ‘pockets’ in her analysis of atmospheres, I explore the generating of ‘atmospheric bubbles’ within the setting. I use the term ‘atmospheric bubbles’ to express the positive often joyful, yet fragile, ephemeral atmospheres that came into being when working with children and young people in waiting areas. Time felt suspended, and the atmosphere had a veil-like quality. The process of engaging with multisensory objects, making with creative materials, and the invitation to be imaginative shaped the ‘atmospheric bubbles’. I typically sat on the floor with participants, surrounded by arts materials and multisensory objects. Objects were shaken, touched, manipulated, and arranged. Drawings and notes were made using scented pens. Materials were examined, selected, placed and glued together to form maps or collages. I worked with participants to photograph what was created and participants described their ideas. These encounters were flexible, responsive, and improvised – there was often a quiet hum of activity as we worked. The interplay of these various modes of expression shaped how the environment felt. These ‘atmospheric bubbles’ were perceptible to others, yet dissipated as quickly as they formed. There was a sense of fragility: once the ‘atmospheric bubble’ formed, the joy, wonder, and creativity that ensued could easily ‘burst’ and be subsumed into another atmosphere. For instance, the child’s name being called out, the buzz of a mobile phone, the rush of staff through the space, and the arrival of a new family were just some of the ways that the atmosphere was disrupted. Staff collecting families for an appointment often joked that they felt like the ‘bad guys’ – this shift was palpable as the bubble ‘burst’ and the child went to their appointment.

Interestingly, some children and young people took sensory objects with them into clinical spaces. There was a sense that the child could retain some of what they had felt within the waiting area, and that what the sensory object(s) enabled (for instance, enjoyment, distraction, intrigue, comfort, although unarticulated) may support them during their appointment(s). In some instances, taking items with them seemed like an act of resistance, both at having to leave the area and at entering the clinical space.

The sub-theme ‘Attuning to atmospheric bubbles’ highlights the potency of ‘things’ and materials that are close to hand, accessible, and self-facilitated. Access to different creative and sensory materials and objects that could be handled, listened to, played with, watched, and manipulated, seemed to promote the unfolding of atmospheres that were absorbing, playful, and distracting within the waiting area.

### Crafting places in the hospital (1.2)

**Figure 7.**
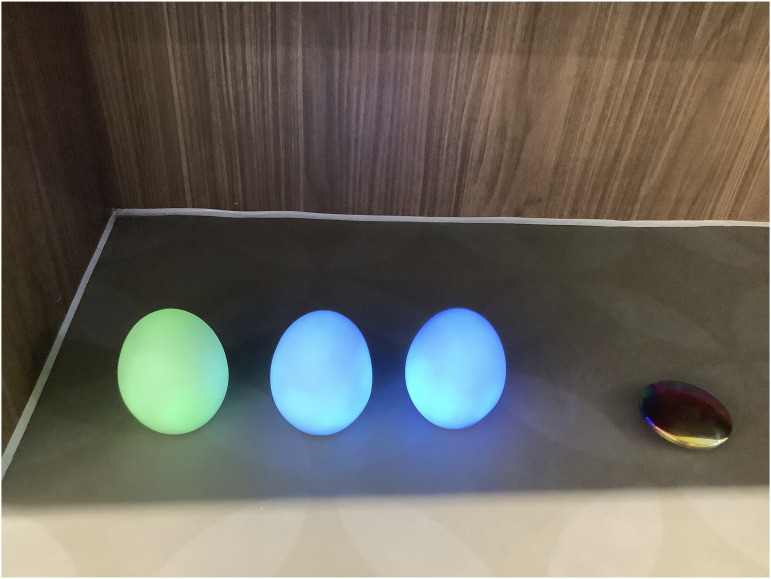
Colour-changing objects left underneath a bench.

‘Crafting places in the hospital’ speaks to how children and young people drew, talked about, and made different places in the hospital to meet their wants and needs. Children’s use of sensory objects demonstrated how they created opportunities for play and escapism in spaces that would typically be unseen or ignored by adults. This use of space was brought to the fore when using the glowing colour-change eggs. Most children and young people sat on the floor to complete the activities, but some younger children crawled under benches in the waiting area. Figure 7 shows objects left behind by a child after taking part in the research.

The child had placed objects underneath the bench, where the full impact of the colour-changing object’s light could be seen. This use of the objects demonstrates how children craft places within the hospital, places that are conducive to them and their needs – sitting or lying under the bench created a more private, den-like place, speaking to the ways in which children perceive areas that would typically be overlooked.

**Figure 8.**
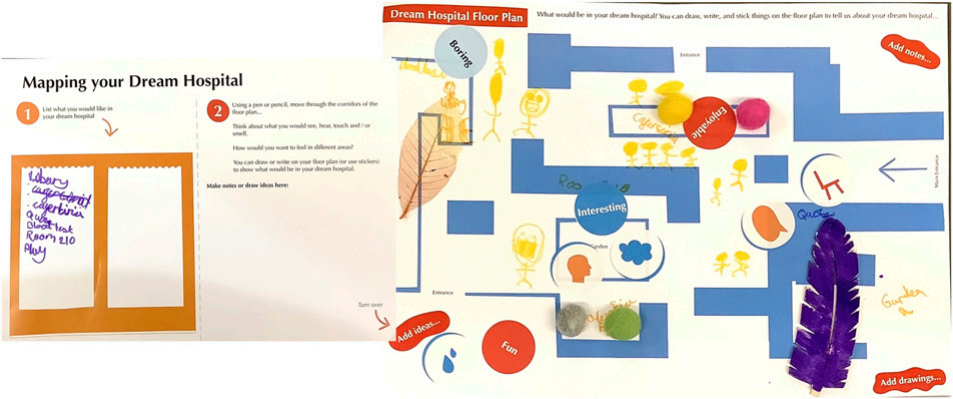
A2025’s map of their dream hospital.

On the crafting of places within the hospital, some young people spoke of creating new places beyond clinical areas. For example, A2025’s map (Figure 8) had an ‘imaginative library’, ‘playful décor’, ‘comfy seating at the main entrance’, and a play area outside the hospital. A2025's careful demarcation of different areas connected with other participants. For example, A2023 described wanting an ‘art room’, ‘a games room’, ‘a dress-up room’, and a room housing ‘fidget toys’, while A2022 spoke of promoting specific feelings within the hospital: they wanted some areas to feel playful and others to feel calm.

The crafting of places within the hospital was apparent when carrying out the research, as running the creative research activities altered spaces. The following reflexive note shares what wheeling the trolley of resources around the hospital felt like and how certain spaces did not feel conducive to carrying out the research.

Wheeling the GOSH Arts trolley into different areas of the hospital seemed to create permission for play, imagination, and creativity. Once at the study building, I tried working in different waiting areas and this included a smaller waiting area – each time this was unsuccessful as I was very much ‘on top of’ families in this area. The space was very narrow and if people didn’t want to take part it was hard to move away and give them room. The use of the trolley gave the research a visibility and staff often expressed intrigue and came over to speak to me about the project. Children were often enthusiastic and wanted to explore the items, yet young people often perceived the research as ‘not for them’. This was challenging to manage and suggested that recruitment could have targeted different age groups on different days, rather than attempt to accommodate everyone at the same time. *Researcher Reflexive Note*

**Figure 9.**
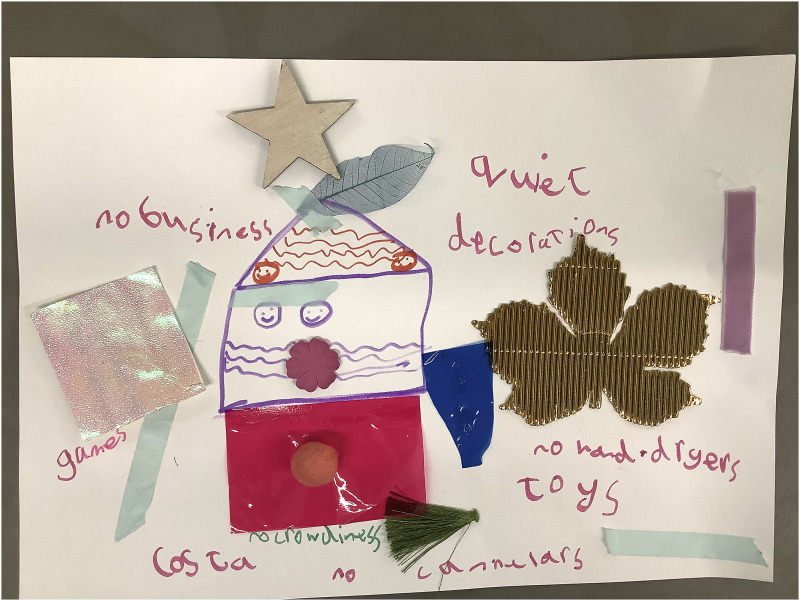
A2029’s collage of their dream hospital.

When considering the creation of places within the hospital some participants drew and wrote down things to omit. For example, A2029 expressed a sensitivity to noise and busy spaces and labelled their collage with instructions such as ‘no business [busyness]’, ‘no hand-dryers’, and ‘no crowdiness [crowds]’ (Figure 9). Some participants referred to limiting clinical objects, for instance, ‘no cannulars [cannulas]’ (A2029), ‘less needels [fewer needles] on show’ (A2027), while A2025 labelled an area for blood tests on their hospital floor plan as ‘boring’. Not all participants spoke to the clinical aspects of the environment, but children picking out things to exclude or hide connects with research around anticipation and movement within the hospital environment (Sumartojo et al., 2020) and demonstrates that children and young people are highly attuned to how different areas of the hospital feel and how sensitive transitioning between different areas can be.

### Creativity and connection (1.3)

As above, the research yielded rich insights. The sub-theme ‘Creativity and connection’ draws upon the research process, and children’s and young people’s wants for the hospital environment. Firstly, the process of taking part in the research and interacting with different sensory items demonstrates the opportunities that certain ‘things’ create for children and young people with hearing and/or visual impairments. The different ways in which children could take part attempted to appeal to potential participants and the inclusion of different sensory items allowed different modes of meaning-making, including embodied, improvised interactions and encounters. As I noted,

I felt nervous about not knowing [what] children’s/young people’s access needs [were], but most parents/guardians were warm and welcoming and would support me if needed. In many instances, interactions consisted of copying movements – passing items between one another, moving them, shaking them, looking through them, and mirroring each other. *Researcher Reflexive Note*

The sensory items and creative materials promoted an outlet within and beyond the research. This was at times challenging but demonstrated children and young people’s specific needs within the waiting areas:

At one point, I was overwhelmed by children who were very energetic and wanted to be loud in their play and to throw the objects. I had not anticipated this or foreseen how I would handle it. I realised that I was beholden to parents/guardians to support this work. In some cases, I think I was perceived as another pair of hands, or someone to provide engagement and potentially ‘respite’ at a difficult time. In these instances, I was not working with the children for the purpose of the research but providing creative engagement – this signifies the outlet/space often needed in the setting. *Researcher Reflexive Note.*

Access to the arts and creative activities were discussed by many participants. For example, A2023 shared how they would repurpose feedback forms so that they could draw while waiting for appointments:

A2023 talked about using the paper and pens provided in wall-mounted display stands to create drawings, as they needed something to do when waiting. They wanted art packs on the walls, like the feedback forms. They also talked about wanting an art room at the entrance or something that is tactile/tactile materials. *Researcher Fieldnote*

**Figure 10.**
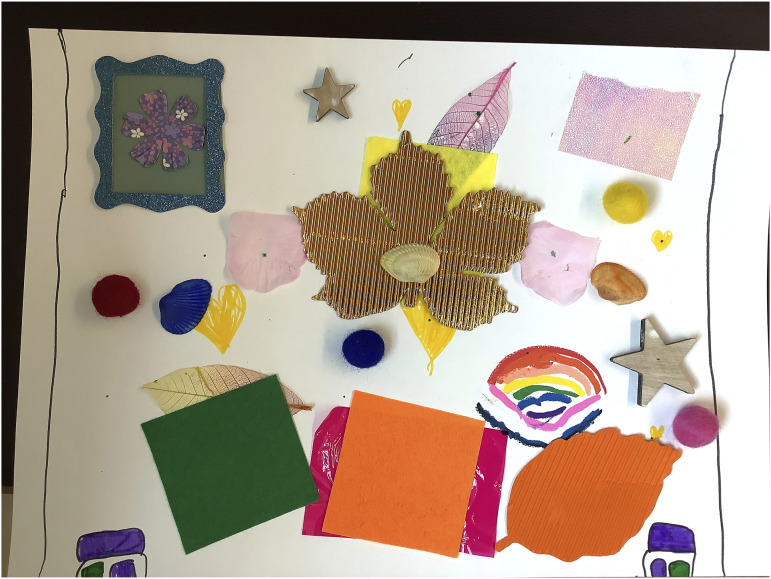
A2023’s collage of their dream hospital.

Several participants spoke of wanting artwork in their dream hospitals, including artwork created by children. For instance, A2027 stated they wanted ‘more vibrant walls’ while A2025 said that they wanted artworks in the consultancy rooms, created by children. A2023 added ‘flower paintings done by children’ to their dream hospital (see Figure 10 and the framed flower in the collage). The importance of creativity and fostering a sense of connection through the arts was thus implicitly expressed.

## Discussion

This study highlighted that children and young people have strong ideas about how a children’s hospital should look and feel, and that embodied, creative, and sensory approaches that are inherently multimodal can lead to a rich process of meaning-making. As Bock (2016) notes, creative flexible ways of working and the ability to choose the mode of expression can support children. The research approaches and the materials used enabled different forms of engagement, and these processes are intimately connected with what this research found (Sakr, 2019). For example, crafting spaces within the hospital was physically apparent through the ways in which children utilised spaces during the research – crawling under benches, taking objects with them into appointments, and ‘taking up space’ by spreading out materials to create workspaces on the floor. Using spaces in this way demonstrates how children can manipulate places to meet their needs (Bock, 2016; Lambert et al., 2014) and utilise areas typically overlooked by adults. This connects with Wohlwend’s (2015) positioning of play as a tactic that sees children work within constraints to create possibilities. This also relates to Potter et al.’s. (2024) work around den-making, which explored children’s experiences of play during the pandemic, finding that children were resourceful and creative, transforming spaces to create a sense of control and autonomy at an uncertain time. Their research, although based in the home, demonstrates how children arranged and used materials and reconfigured spaces in meaningful ways. The creation of den-like places within the hospital could similarly be said to promote a sense of comfort, privacy, and control at an uncertain/difficult time. This use of space was specific to children and was not undertaken by young people, speaking to the need to attend to different ages and abilities within the hospital (Birch et al., 2007).

This research found that children and young people alike wanted art integrated within the hospital. Interestingly, the study site was home to artworks carefully embedded within the architecture of the building, but these pieces were not discussed during the research. This may have been due to the aspirational focus of the research (i.e., the emphasis on participants’ dream hospital environments), yet it may also speak to a need to ‘activate’ the artworks to encourage children and young people to interact with them. Children and young people discussed wanting art created by children in the hospital and there was a sense that this could act as a vehicle for connection. The relational aspect of art within the environment is something increasingly explored, as is the power of the arts in children’s healthcare settings (Belver and Ullán, 2010; Bishop, 2017; Ullán and Belver, 2021). The work of GOSH Arts involves a participatory arts programme and the commissioning and integration of artworks within the built environment. The programme is renowned for involving children and young people. Participants expressed want for children and young people’s artworks to be seen within the environment suggests greater opportunities to share stories of participatory projects so that patients are aware of how existing works come into being. The want to have artworks created by children and young people also speaks to a need for connection with others, and to feel represented within the hospital environment. Temporary exhibitions, sharing artworks through digital technologies, and interactive artworks could meet this desire for connection.

The findings demonstrate how creative opportunities can be promoted, through for instance, providing accessible materials and multisensory objects that cater for a range of different ages and different access needs, including promoting and supporting self-initiated/facilitated creativity. For example, the research process saw many children and young people take resources around the hospital with them, while A2023’s use of feedback forms suggests the possibility of installing self-facilitated wall-mounted resources around the hospital. The want for such provision connects with a hackathon (design competition) (Kirsten et al., 2015) which brought together young designers and young cancer patients to design ‘a youth-friendly ward environment for young people with cancer’. The concept of a ‘youth box’ was selected as the favourite design. This acted as a welcome package for new patients and contained materials such as notebooks and pens, inspirational quotations from patients, earplugs, pamphlets, and information about activities. The provision of activity resources is not new within hospital arts programmes (see e.g. GOSH Arts, n.d; The Children’s Hospital Charity, 2020) and the pandemic saw an increasing number of programmes pivot to provide accessible non-facilitated arts resources e.g., artists produced downloadable resource sheets. Nevertheless, the space and resource required to install, for example, wall-mounted arts resources or to distribute packs across the hospital is not easily found. Hospital arts teams are often under-resourced and under-staffed and so their capacity to commission, distribute, and maintain materials is limited. An outcome from this project was the commission and installation of the artwork ‘Murmuration’, created for and with young people at GOSH. The artwork, created by Gawain Hewitt, contains digital changeable elements, and connects to a series of online musical instruments (Murmuration, n.d) so that young people can create music both in and beyond the hospital. The artwork seeks to promote a sense of connection between young people in the hospital and was created through participatory work with GOSH patients, GOSH YPAG, and GOSH’s Young People’s Forum.

The attention to atmospheres within this work speaks to how exploring the hospital environment through material, sensory, creative, and embodied means can promote understanding hospital environments in alternative ways, to better understand how settings can support children and young people. Much like Ullán et al. (2012), this research found that children and young people are highly attuned to the different atmospheres that form within the hospital. This research aligns with the work of McLaughlan and Willis (2021), Sumartojo and Pink (2018) and Sumartojo et al. (2020) to call for attention to atmospheres when considering enhancing hospital environments. Atmospheres are unfixed and ever-changing, and promoting opportunities for creative, imaginative, and sensory encounters can transform how areas feel for children and young people.

## Conclusion

This research demonstrates how children and young people with varying sensory and access needs can be supported to create and share what is important to them within the hospital. Findings demonstrate the opportunities that multiple modes of expression afford in understanding children and young people’s use of spaces within the hospital, illuminating how the environment feels, and understanding the importance of creativity within the hospital. Future research could explore the longstanding relationships that children and young outpatients often have with the hospital and embrace this within the research process. For instance, research approaches could be adapted to gather insights over time, so that outpatients could be involved over a prolonged period. This could allow for exploration of ideas around the unfixed nature of the hospital environment. Such adaptations may appeal to young people and allow us to better understand what is important to them within these environments. This research used multiple approaches to promote inclusivity, but this was at times challenging to realise in practice (as detailed within the researcher reflexive notes). Future research could be developed to work with specific groups of outpatients (e.g. specific age ranges, specific access needs) to better support accessibility and engagement.

## Data Availability

Please note that data from the Sensing Spaces of Healthcare project is not available due to ethical restrictions.
